# Mycoplasma Suppression of THP-1 Cell TLR Responses Is Corrected with Antibiotics

**DOI:** 10.1371/journal.pone.0009900

**Published:** 2010-03-25

**Authors:** Ekaterina Zakharova, Jaykumar Grandhi, Mark D. Wewers, Mikhail A. Gavrilin

**Affiliations:** Division of Pulmonary Allergy Critical Care and Sleep Medicine, Davis Heart and Lung Research Institute, The Ohio State University, Columbus, Ohio, United States of America; CNRS/Université de Toulouse, France

## Abstract

Mycoplasma contamination of cultured cell lines is a serious problem in research, altering cellular response to different stimuli thus compromising experimental results. We found that chronic mycoplasma contamination of THP-1 cells suppresses responses of THP-1 cells to TLR stimuli. For example, *E. coli* LPS induced IL-1 β was suppressed by 6 fold and IL-8 by 10 fold in mycoplasma positive THP-1 cells. Responses to live *F. novicida* challenge were suppressed by 50-fold and 40-fold respectively for IL-1β and IL-8. Basal TLR4 expression level in THP-1 cells was decreased by mycoplasma by 2.4-fold (p = 0.0003). Importantly, cell responses to pathogen associated molecular patterns are completely restored by mycoplasma clearance with Plasmocin. Thus, routine screening of cell lines for mycoplasma is important for the maintenance of reliable experimental data and contaminated cell lines can be restored to their baseline function with antibiotic clearance of mycoplasma.

## Introduction

Mycoplasma is a filterable bacteria which does not have a rigid cell wall and is very resistant to a great number of antibiotics [1]. Mycoplasmal growth is rapid; and contamination of cultured human cells by mycoplasma is a significant problem in experimental research. Our study indicates that chronic mycoplasma contamination of THP-1 cell cultures interferes with Toll-like receptor (TLR) functions in response to a variety of pathogens and toxins. Toll-like receptors (TLR) are pattern-recognition receptors which affect gene transcription in response to a pathogen associated molecular patterns (PAMP) like bacterial lipopolysaccharide (LPS) [2], [3]. TLR stimulation initiates a signal transduction pathway which leads to the secretion of pro-inflammatory cytokines such as IL-8 and IL-1β [4], [5]. Many researchers report that mycoplasmal PAMPs, like lipoproteins induce an inflammatory response in primary monocytes and THP-1 cells in a way similar to LPS [6], [7], [8]. However, we show that chronic contamination of THP-1 cells by mycoplasma suppresses the pro-inflammatory cellular response to *E. coli* LPS and *Francisella novici*da, and that this suppression can be corrected with antibiotic clearance of mycoplasma.

## Materials and Methods

Human monocytic THP-1 cell lines were cultured in RPMI 1640 (MediaTech, Inc) containing 10% fetal bovine serum (endotoxin-free; Atlanta Biologicals) and 1% penicillin-streptomycin solution (Invitrogen Life Technologies). Mycoplasma-positive and negative THP-1 cells were stimulated either with *E. coli* LPS (100 ng/ml) or *Francisella novicida* (strain JSG2401; U112) for 15 hours in antibiotics-free media. The release of IL-1β and IL-8 in the culture media was evaluated by ELISA developed in our laboratory [9]. RNA was extracted using Trizol (Invitrogen Life Technologies) and evaluated for gene expression using RT-qPCR as described previously [10]. Briefly, the expression of the genes of interest was calculated in relative copy numbers (RCN), which is relative to the average expression value of two housekeeping genes. Data were analyzed within one group and between two groups using paired t-test. For all analyses, *p*<0.05 was considered to be significant. To check for mycoplasmal contamination, genomic DNA was isolated using a Puregen kit (Qiagen; Puregen Accessories), and verified by PCR where a high quality genomic DNA is an important factor. The combination of the following three sets of primers allowed detection of most mycoplasma species [11], [12], [13]: Myco-280 forward 5′- gggagaaacaggattagataccct-3′ and reverse 5′-tgcaccatctgtcactctgttaacctc-3′; Myco-500 forward 5′-ggcgaatgggtgagtaacacg-3′ and reverse; and Myco-717 forward F: 5′-actcctacgggaggcagcagta-3′ and reverse 5′-tgcaccatctgtcactctgtt-3′. PCR conditions were 5 min 95°C and then 35 cycles at 94°C for 15 s, at 60°C for 15 sec, and at 72 for °C for 15 sec.

To eliminate mycoplasma contamination, mycoplasma positive THP-1 cells were treated with 25 µg/ml of Plasmocin (InvivoGen) for three weeks. Aliquots were taken every three to four days for detection of mycoplasma by PCR. When cells were confirmed to be a mycoplasma negative, they were further maintained in RPMI 1640 media in the absence of Plasmocin.

## Results and Discussion

**Figure 1:**
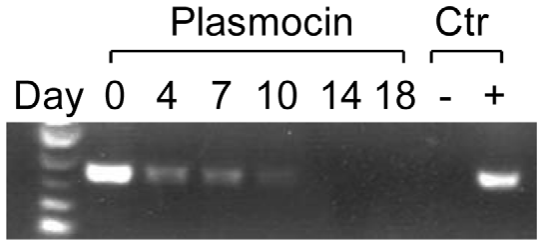
Plasmocin-dependent removal of mycoplasmal contamination of THP-1 cells. THP-1 cells, chronically infected with mycoplasma, were treated with Plasmocin (25 µg/ml every 3 days) and aliquots were taken every three to four days for detection of mycoplasma by PCR. DNA from mycoplasma negative (−) and positive (+) cells was served as control (Ctr).

**Figure 2:**
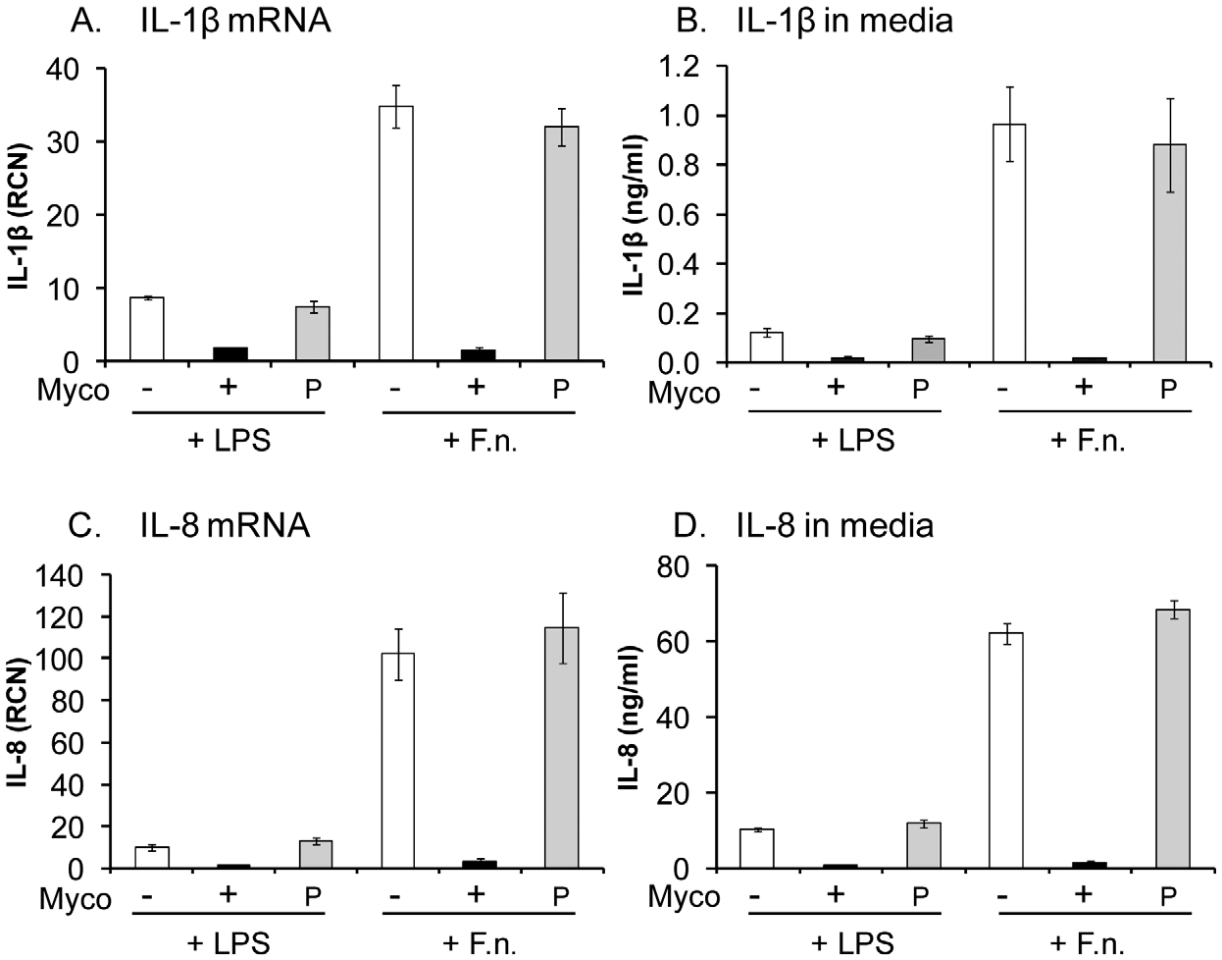
Chronic mycoplasma infection inhibits THP-1 cells response to PAMPs. THP-1 cells, mycoplasma negative (−), positive (+) or treated with Plasmocin and confirmed to be a mycoplasma negative (P) were stimulated either with *E. coli* LPS or *F. novicida* (F.n.) for 15 hours in antibiotic-free media. Gene expression was measured in relative copy numbers (RCN) and cytokine concentration in ng/ml. Mycoplasma positive cells show decrease in expression of IL-1β message (A) and mature cytokine release (B), as well as IL-8 mRNA expression (C) and protein release (D). Mycoplasma removal restores cellular function to these PAMPs (P). Data are expressed as mean ± SEM. n = 4 for qPCR of THP-1 treated with LPS and n = 8 for all other samples.

**Figure 3:**
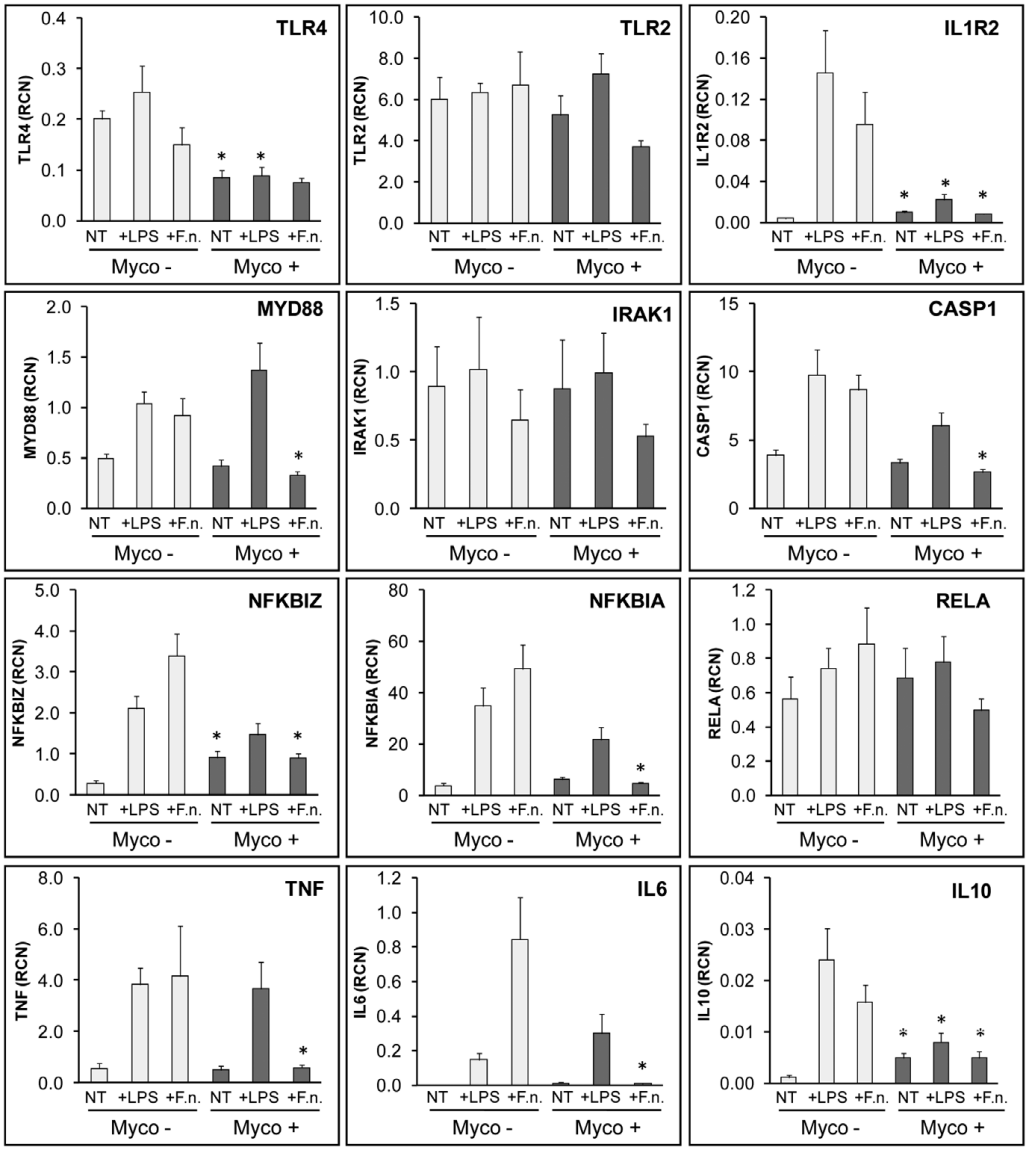
Gene expression in mycoplasma negative and positive THP-1 cells stimulated with LPS or *F. novicida*. Mycoplasma negative (Myco −) and positive (Myco +) THP-1 cells were stimulated either with *E. coli* LPS or *F. novicida* (F.n.) for 15 hours in antibiotic-free media. Gene expression was measured in relative copy numbers (RCN). Data are expressed as mean ± SEM. n = 6. Asterisk (*) represents a *p* value<0.05 in comparison between two groups, to show significance of mycoplasma contamination.

Some THP-1 cell passages from our laboratory showed no response to TLR agonists like *E. coli* LPS, or *F. novicida*. To determine the mechanism for this anergy, we tested whether these cells were mycoplasma contaminated by analyzing the cells for mycoplasma DNA by PCR. We found that cells which showed minimal responses in IL-1β or IL-8 release after addition of LPS or *F. novicida* tested positive for mycoplasma. Because we had created a number of unique THP-1 cells stably expressing various genes, we were interested in removal of mycoplasma rather than discarding those cell lines. Treatment of mycoplasma positive cells with Plasmocin effectively removed the presence of mycoplasma within 14 days (Figure 1). To determine if the mycoplasma contamination was responsible for the anergy, we treated mycoplasma negative THP-1 cells (from ATCC, lot 385653, or plasmocin-treated from our stock) and mycoplasma positive THP-1 cells with LPS or *F. novicida*, and compared their IL-1β and IL-8 RNA message expression and cytokines release in cell culture media (Figure 2). We found that mycoplasma positive cells showed significant decrease in mRNA expression in response to both LPS and *F. novicida* (Figure 2). In contrast, removal of mycoplasma with Plasmocin rendered samples fully responsive to LPS and *F. novicida* (Figure 2). Since it is known that *F. novicida* is detected by TLR2 [14] and LPS is sensed by TLR4 [15], so it is likely that chronic mycoplasma infection affects TLR2 and TLR4 signaling pathways. In part, we tested such possibility by screening gene expression patterns in mycoplasma negative and positive cells. As shown in Figure 3, mycoplasma affects baseline gene expression for *TLR4, IL1R2, IKB1Z*, and *IL10*. Furthermore, LPS stimulation of mycoplasma positive THP-1 cells showed significant suppression of the *TLR4*, *IL1R2*, and *IL10* genes as compared to controls (Figure 3). The effect was even more pronounced to *Francusella* challenge affecting *IL1R2, MYD88, NFKBIZ, NFKBIA, CASP1, TNF, IL10, and IL6* significantly. There was no difference between mycoplasma negative and positive THP-1 cells *for TLR2, RELA* and *IRAK1* genes expression (Figure 3).

Numerous reports have shown that mycoplasmal PAMPs stimulate TLR-mediated pro-inflammatory response [7], [8]. This is different from our observation and may be explained by the phenomenon of “LPS tolerance”– the transient unresponsiveness of cells to LPS after repeated or prolonged stimulation with LPS [16]. The effects of LPS tolerance include the inhibition of TLR signaling by inducible negative regulators, the suppression of production of pro-inflammatory cytokines such as TNF-α, IL-1β and IL-8, and alterations of the TLR signaling complex [17]. LPS tolerance has traditionally been viewed as a hypo-responsive state of macrophages resulting from receptor desensitization [16]. Several groups have reported examples of pro- and anti-inflammatory gene induction in tolerant macrophages [18], [19], and others have reported that pre-incubation with low doses of LPS can cause changes in the intracellular signal transduction pathway which may lead to hyposensitivity and LPS tolerance [20]. In contrast, it has also been reported that the expression of IL-1α and β mRNA is markedly reduced in cell cultures contaminated by mycoplasma, which corresponds to our observations [1]. LPS tolerance may also be induced by mycoplasmal lipopeptides that impair the MyD88-depending signaling by inhibiting LPS-mediated activation of IL-1 receptor-associated kinase (IRAK1) [21].

Of course the best way to eliminate mycoplasma contamination is to discard contaminated cells. However, in some cases a valuable cell line may require a treatment of mycoplasma contamination to restore functionality. Our data show that chronic mycoplasma infections suppress TLR function and that cell treatment with Plasmocin (a combination of two bactericidal compounds) can effectively remove mycoplasma contamination (as detected by PCR) and restore the ability of cells to respond to PAMPs.
